# Clinical Low-Dose Photon-Counting CT for the Detection of Urolithiasis: Radiation Dose Reduction Is Possible without Compromising Image Quality

**DOI:** 10.3390/diagnostics13030458

**Published:** 2023-01-26

**Authors:** Julius Henning Niehoff, Alexandra Fiona Carmichael, Matthias Michael Woeltjen, Jan Boriesosdick, Arwed Elias Michael, Bernhard Schmidt, Christoph Panknin, Thomas G. Flohr, Iram Shahzadi, Hansjuergen Piechota, Jan Borggrefe, Jan Robert Kroeger

**Affiliations:** 1 Department of Radiology, Neuroradiology and Nuclear Medicine, Johannes Wesling University Hospital, Ruhr University Bochum, 44801 Bochum, Germany; 2 Department of Urology, Johannes Wesling University Hospital, Ruhr University Bochum, 44801 Bochum, Germany; 3 Siemens Healthcare GmbH, 91052 Erlangen, Germany

**Keywords:** photon-counting CT, low-dose CT, CT image quality, CT radiation dose, urolithiasis

## Abstract

**Background:** This study evaluated the feasibility of reducing the radiation dose in abdominal imaging of urolithiasis with a clinical photon-counting CT (PCCT) by gradually lowering the image quality level (IQL) without compromising the image quality and diagnostic value. **Methods:** Ninety-eight PCCT examinations using either IQL70 (n = 31), IQL60 (n = 31) or IQL50 (n = 36) were retrospectively included. Parameters for the radiation dose and the quantitative image quality were analyzed. Qualitative image quality, presence of urolithiasis and diagnostic confidence were rated. **Results:** Lowering the IQL from 70 to 50 led to a significant decrease (22.8%) in the size-specific dose estimate (SSDE, IQL70 4.57 ± 0.84 mGy, IQL50 3.53 ± 0.70 mGy, *p* < 0.001). Simultaneously, lowering the IQL led to a minimal deterioration of the quantitative quality, e.g., image noise increased from 9.13 ± 1.99 (IQL70) to 9.91 ± 1.77 (IQL50, *p* = 0.248). Radiologists did not notice major changes in the image quality throughout the IQLs. Detection rates of urolithiasis (91.3–100%) did not differ markedly. Diagnostic confidence was high and not influenced by the IQL. **Conclusions:** Adjusting the PCCT scan protocol by lowering the IQL can significantly reduce the radiation dose without significant impairment of the image quality. The detection rate and diagnostic confidence are not impaired by using an ultra-low-dose PCCT scan protocol.

## 1. Introduction

Kidney and ureter stones can cause severe clinical symptoms leading to hospitalization, renal impairment and death. Non-contrast CT scans are usually the preferred cross-sectional imaging method for the detection of calculi occluding the urinary tract. The advantages of non-contrast CT scans are the high sensitivity and specificity for the detection of urolithiasis [1,2]. A major disadvantage, however, is that CT examinations always require exposure to ionizing radiation.

Overall, the prevalence as well as the incidence of urolithiasis have increased over the past few decades [3,4,5]. Although the highest prevalence is observed in elderly patients, kidney and ureter stones can also occur in young patients [4]. Taking into account the high recurrence rate, it is not unlikely that these patients will undergo several CT scans during their lifetimes [3,6]. Thus, developing and optimizing CT scan protocols with reduced radiation doses are crucial.

Various techniques to reduce radiation doses have been employed and different low-dose CT scan protocols have been successfully established in clinical routine; e.g., CT protocols with reduced tube charge current, increased pitch factor, additional tin filter or iterative reconstruction algorithms [7,8,9,10,11,12,13,14].

An entirely new technology designed to reduce the radiation dosage was recently introduced with the first clinical photon-counting CT (PCCT) scanner. These CT scanners make use of a newly developed photon-counting detector (PCD) that consists of a single thick layer of a semiconductor material. Unlike conventional energy-integrating detectors (EID), PCDs convert incoming X-rays directly into an electrical signal and do not require additional conversion steps from X-ray to visible light and from visible light to an electrical signal [15,16].

An initial study compared the radiation dose parameters as well as the image quality of the novel PCCT and a conventional EID CT when using the clinical scan protocol of each CT scanner for the detection of urolithiasis. It showed that the PCCT required a significantly lower radiation dose (approximately 30%) to provide significantly better image quality, thus leading to an excellent detection rate of kidney and ureter stones as well as to a high diagnostic confidence of the radiologists evaluating the CT scans [17].

The purpose of the present study is to evaluate the feasibility of reducing the radiation dose of the low-dose scan protocol for the detection of urolithiasis by gradually lowering the image quality level (IQL). The impact on image quality, diagnostic performance and diagnostic confidence as well as the influence on measurements of the size of ureter stones of the reduced radiation dose protocols are evaluated.

## 2. Material and Methods

### 2.1. Patient Population

This study was approved by the institutional review board. Patient consent was waived due to the retrospective study design. In total, low-dose CT examinations of 98 consecutive patients with clinical suspicion of urolithiasis were retrospectively included in this study. The CT scans were performed between September 2021 and March 2022. Patients included in this study were not preselected (e.g., regarding weight, age, or gender).

### 2.2. CT Protocols and Image Acquisition

All CT examinations were performed in a supine position. Scan parameters for all examinations with the PCCT (NAEOTOM Alpha, software version syngo CT VA40, Siemens Healthineers, Erlangen, Germany) were as follows: tube voltage 100 kVp, tin filter, detector configuration 144 × 0.4 mm, pitch 0.6 and gantry rotation time 0.5 s.

All CT scan protocols compared in this study used automatic tube current modulation techniques, but at different dose levels, which was achieved by adjusting the so-called “image quality level” (IQL) parameter. The IQL is a parameter developed to provide a CT system independent definition of image quality. It represents quality reference milliampere-seconds (mAs)—a previously defined parameter—denoting the effective mAs applied to the patient based on protocol- and patient-specific attenuation information to produce a desired image quality—additionally being corrected according to detector and CT geometry-specific properties. The IQL was initially set at level 70 (N = 31 CT scans), then lowered to level 60 (N = 31 CT scans) and ultimately lowered to level 50 (N = 36 CT scans).

The reconstruction parameters were as follows: slice thickness 2 mm, slice increment 1 mm, image matrix 512 × 512, soft tissue kernel (Br36) and quantum iterative reconstruction (QIR) level Q4.

### 2.3. Radiation Dose

The analysis included the following parameters: Computed tomography dose index (CTDI), dose length product (DLP), effective dose (ED, calculated as explained by Stamm et al.) and the size-specific dose estimate (SSDE) [18]. Subgroups were formed with regard to gender and body mass index (BMI).

### 2.4. Quantitative Image Analysis

Regions of interest (ROI), constant in size (diameter 1 cm) on all images, were drawn in the subcutaneous fat and the paravertebral muscle. Image noise was defined as standard deviation (SD) of the mean density (in Hounsfield Units, HU) of each ROI. Signal-to-noise ratio (SNR) was defined as mean density (HU) divided by the SD of each ROI.

### 2.5. Qualitative Image Analysis

CT images were evaluated independently by 3 radiologists with at least 2 years of experience. Overall image quality, image noise and image sharpness were rated on a 5-point Likert scale (5 = very good image quality/very little noise/perfectly defined structures with very sharp contours; 1 = very poor image quality/too much noise for evaluation/contours are blurred, images are inadequate for diagnostic reporting). 

In addition, the radiologists screened each CT scan for the presence of kidney or ureter stones and rated their diagnostic confidence on a 5-point Likert scale (5 = very confident; 1 = not confident).

While evaluating, readers were not aware of the CT scan protocol that was used to acquire the particular CT images. Cases were presented in a randomized order. Ground truth was based on the radiology report and expert consensus reading in discordant cases.

### 2.6. Stone Size Measurements

In total, the largest axial diameter of 45 ureter stones (15 ureter stones on each IQL) was measured independently by 6 radiologists using the exact same image settings (e.g., reconstruction kernel, slice thickness, QIR level and window settings). The interreader reliability was calculated for each IQL.

### 2.7. Statistical Analysis

Statistical analysis was performed with established software packages (SPSS Statistics 28, IBM, Armonk, New York, NY, USA/Excel 2016, Microsoft, Redmond, Washington, DC, USA/R Core Team (2021), R: A language and environment for statistical computing, R Foundation for Statistical Computing, Vienna, Austria, RStudio Version 1.4.1106). If not stated otherwise, all data are presented as mean ± SD.

Differences between groups with continuous variables were evaluated using a one-way ANOVA. In case significant differences were found, pair-wise Bonferroni post hoc tests were performed. For ordinal variables and subgroup analysis (to account for the smaller sample size), non-parametric statistics were used. Kruskal–Wallis test by ranks was used to test for differences between groups, and in case of significant differences, post hoc pair-wise Mann–Whitney test with Bonferroni correction was used. Kendall’s Coefficient of Concordance (*W*) was calculated to assess the interreader reliability of the qualitative image analysis. Two-way mixed intra-class correlation coefficient was calculated to assess interreader reliability of stone size measurements for the different dose groups. Differences in terms of detection rates were tested with the Fisher’s exact test for statistical significance. *p*-values < 0.05 were considered indicative of statistical significance.

## 3. Results

### 3.1. Patient Population

In total, low-dose CT examinations of 98 patients were included in the analysis. Thirty-one patients (mean age 51 years, range 21–94 years) were examined using the IQL 70. Likewise, thirty-one patients (mean age 49 years, range 19–88 years) were examined using the IQL 60. Thirty-six patients (mean age 55 years, range 19–86 years) were examined using the IQL 50. The distribution of the patients in terms of gender and BMI is shown in Table 1. Mean BMI was highest in the patient group scanned with IQL 50 and lowest in the patient group scanned with IQL 60. Differences in BMI, however, were not statistically significant.

### 3.2. Radiation Dose

**Figure 1:**
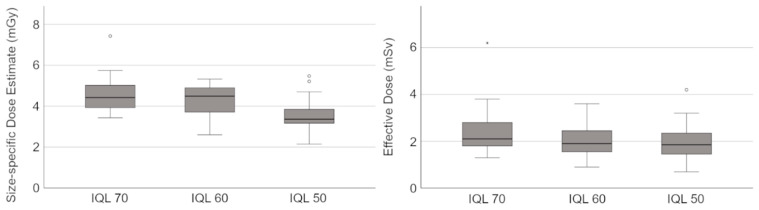
Box plots showing the size-specific dose estimate (SSDE) and the effective dose (ED) of CT scans using the image quality levels (IQLs) 70, 60 and 50. SSDE and ED differed significantly between the IQLs (see also Table 2).

CTDI_vol_, DLP, ED and SSDE are shown in Table 2 and Figure 1. Subgroups were formed with regard to gender and BMI.

In total, lowering the IQL from 70 to 50 led to a significant decrease (22.8%) of the SSDE (IQL 70: 4.57 ± 0.84 mGy vs. IQL 50: 3.53 ± 0.70 mGy, *p* < 0.001). In addition, when considering each individual subgroup, there was a significant reduction in the SSDE between IQL 70 and IQL 50 (see Table 2). The greatest difference in SSDE between IQL 70 and IQL 50 was observed among patients with a BMI > 30 kg/m^2^ (IQL 70: 5.32 ± 1.04 mGy vs. IQL 50: 3.91 ± 0.54 mGy, *p* = 0.004).

In total, the ED decreased by about 18.9% between IQL 70 (2.43 ± 0.99 mSv) and IQL 50 (1.97 ± 0.72 mSv, *p* = 0.063). The difference in ED between IQL 60 (2.02 ± 0.67 mSv) and IQL 50 (1.97 ± 0.72 mSv, *p* = 1.000), however, is comparatively small. Similar to the SSDE, the greatest difference in ED between IQL 70 and IQL 50 was observed among patients with a BMI > 30 kg/m^2^ (IQL 70: 3.33 ± 1.36 mSv vs. IQL 50: 2.54 ± 0.76 mSv).

### 3.3. Quantitative Image Analysis

**Figure 2:**
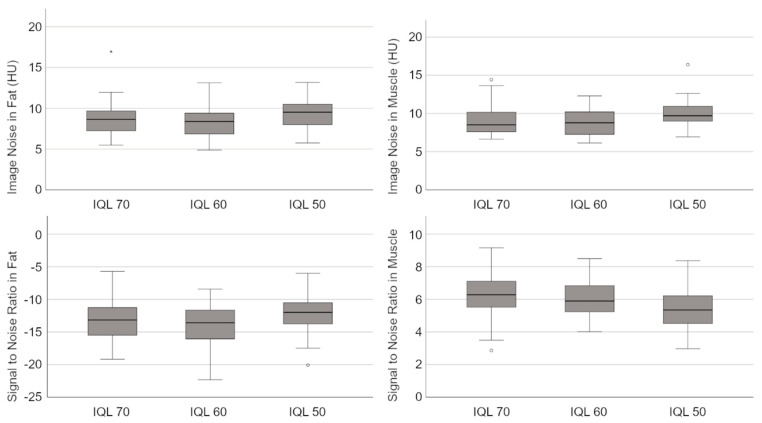
Box plots showing the signal-to-noise ratio (SNR) and the image noise measured in muscular tissue and subcutaneous fat of CT scans using the image quality levels (IQLs) 70, 60 and 50.

**Figure 3:**
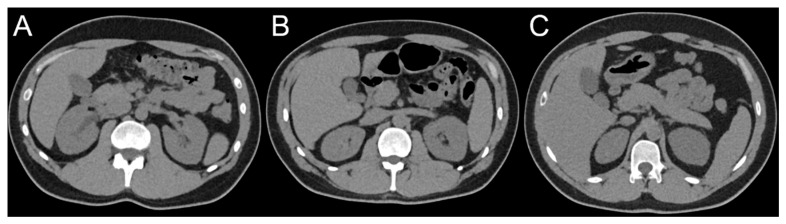
Examples of CT scans using the image quality levels (IQLs) 70 (**A**), 60 (**B**) and 50 (**C**).

The quantitative parameters describing the image quality (SNR, image noise) are shown in Table 3 and Figure 2. Examples of CT scans performed with different IQLs are presented in Figure 3.

Lowering the IQL from 70 to 50 led to a minimal increase in the image noise in muscular as well as in adipose tissue (e.g., in muscle, IQL 70: 9.13 ± 1.99 vs. IQL 50: 9.91 ± 1.77, *p* = 0.248). The increase of the image noise was not statistically significant. At the same time, the SNR decreased in muscular tissue as well as in subcutaneous fat. The decrease in the SNR in muscular tissue was statistically significant (IQL 70: 6.20 ± 1.52 vs. IQL 50: 5.39 ± 1.25, *p* = 0.044).

### 3.4. Qualitative Image Analysis

The results of the qualitative image analysis are shown in Table 4. 

Taking all CT scans using the IQL 70 into account, there were three false negative and three false positive findings among all three radiologists, which translates to a detection rate of 94.7%. The readings of CT scans using the IQL 60 contained six false negative and two false positive findings, leading to a detection rate of 91.3%. When considering the CT scans acquired with the IQL 50, all three radiologists had a detection rate of 100% with no false negative and altogether three false positives. There were no significant differences between detection rates when comparing IQLs 70 and 50 (*p* = 0.097) or IQLs 70 and 60 (*p* = 0.511). The difference in detection rates of IQLs 60 and 50 was statistically significant (*p* = 0.028).

The diagnostic confidence reported by the radiologists was at a very high level and did not differ significantly (*p* = 0.653) between IQL 70 (mean 4.44 ± 0.91), IQL 60 (mean 4.48 ± 0.94) and IQL 50 (mean 4.47 ± 0.83). Likewise, the overall image quality, image noise and image sharpness, as rated by the radiologists, only showed minor differences between IQLs 70, 60 and 50 that were not statistically significant; e.g., the mean rating of the overall image quality was 4.19 ± 0.86 on IQL 70, 4.28 ± 0.94 on IQL 60 and 4.07 ± 1.00 on IQL 50 (*p* = 0.311). The interreader reliability of the qualitative image analysis was high for all criteria (*W*: overall image quality = 0.83, image noise = 0.76, image sharpness = 0.79).

### 3.5. Stone Size Measurements

**Figure 4:**
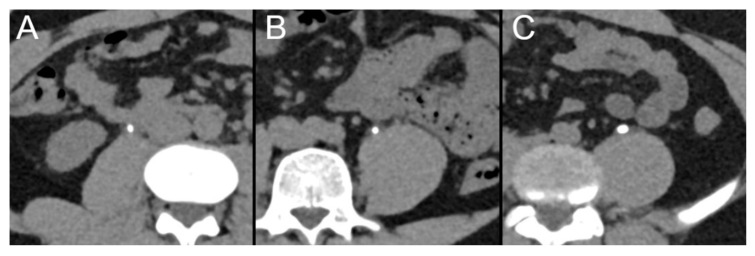
Examples of ureter stones that were included in the analysis of the stone size measurements. CT scans were performed with image quality levels (IQLs) 70 (**A**), 60 (**B**) and 50 (**C**).

In total, 45 ureter stones (15 ureter stones at each IQL) were measured independently by six radiologists (R1–R6) using the exact same image settings (reconstruction kernel, slice thickness, window settings; see Figure 4). Radiologists measured mean stone sizes of 4.2 ± 2.0 mm (R1), 4.3 ± 2.0 mm (R2), 4.8 ± 2.0 mm (R3), 4.2 ± 1.7 mm (R4), 4.3 ± 2.0 mm (R5) and 4.4 ± 2.0 mm (R6). Intra-class correlation coefficients were similar between the IQLs (IQL 70 = 0.995, IQL 60 = 0.994, IQL 50 = 0.994).

## 4. Discussion

The purpose of the present study was the evaluation of radiation dose parameters as well as the image quality achieved with the low-dose PCCT scan protocol for the detection of urolithiasis after gradually lowering the IQL. In addition, we evaluated the impact of the adjusted scan protocol on the detection rate and diagnostic confidence of radiologists as well as the influence on measurements of the size of ureter stones.

Overall, lowering the IQL led to significantly lower radiation doses. However, certain aspects need to be taken into account when considering individual parameters. When looking at the EDs of all patients, it was noticeable that there were only small differences between the individual IQLs; e.g., 0.05 mSv difference between IQL 60 and IQL 50. This is due to the fact that the calculation of the ED does not consider the patients´ body sizes. Although there were no significant differences in terms of BMI between the patient groups, the average BMI of the patients examined with IQL 50 (BMI 28.85 ± 6.19 kg/m^2^) was still markedly higher compared to the other patient groups (e.g., IQL 60: 25.89 ± 5.10 kg/m^2^). When looking at the subgroups including only patients with a BMI < 30 kg/m^2^, there is, as expected, a steady decrease in the ED from 2.12 ± 0.58 mSv on IQL 70 to 1.65 ± 0.46 mSv on IQL 50 (*p* = 0.023).

Due to the differences in mean BMI between the patient groups, the SSDE that additionally considers the body diameter is possibly more reasonable for quantifying the reduction in radiation doses between IQLs. The SSDE decreased steadily and significantly from 4.57 ± 0.84 mGy on IQL 70 to 3.53 ± 0.70 mGy on IQL 50 (*p* < 0.001). Simultaneously, significant differences in the SSDE were found when comparing each individual subgroup.

An interesting observation of this study concerns the subgroups of patients with a BMI > 30 kg/m^2^. When comparing these patients, the reduction in ED as well as SSDE between IQL 70 and IQL 50 was the greatest. Due to the small number of patients with a BMI > 30 kg/m^2^ in this study, it would be inappropriate to draw general conclusions from this observation. However, it can possibly be taken as an indication for the potential of the PCCT for reducing radiation doses in obese patients.

The comparison of the radiation doses described in the present study with radiation doses of previous studies is difficult due to differing scan protocols. However, the definitions for the terms “low dose” and “ultra-low dose” described in the literature can possibly serve as a guide. Rob et al., for example, reporting on a systematic review about non-contrast CT scans for the detection of urolithiasis, defined EDs of ≤3.5 mSv as “low dose” and ≤1.9 mSv as “ultra low dose” [19]. Other studies considered EDs of <4 mSv to be “low dose” [2,20,21]. Based on these definitions, the scan protocols described in the present study can surely be considered as “low dose”. The EDs used in certain subgroups (male patients, BMI < 30 kg/m^2^ on IQL 50) may even be considered as “ultra-low dose”.

The qualitative as well as the quantitative image quality assessment yielded similar results. Lowering the IQL from 70 to 50 led to a slight increase in the image noise and a slight decrease in the SNR. However, these changes were minimal and in most cases statistically not significant. Likewise, the radiologists evaluating the CT images did not notice any major changes in the image quality throughout the IQLs. The detection rate of kidney and ureter stones did not differ markedly between the IQLs and was highest on the lowest IQL. Diagnostic confidence as rated by the radiologists was high and not influenced by the IQL. Furthermore, lowering the IQL did not influence the measurements of the size of ureter stones. The intra-class correlation coefficients were excellent throughout all IQLs.

The present study has certain limitations. Although there were no significant differences, the mean BMI of some patient groups differed markedly, which influenced the applied radiation doses. Forming subgroups and calculating the SSDE can help to overcome this issue. However, this in turn means that the number of patients in some subgroups is relatively low. Thus, no general conclusions about the potential to reduce radiation doses in obese patients can be drawn.

## 5. Conclusions

In conclusion, adjusting the PCCT scan protocol by lowering the IQL can significantly reduce the applied radiation dose for patients without significant impairment of the image quality. The detection rate as well as diagnostic confidence are not negatively influenced by using an (ultra-) low-dose PCCT scan protocol. The results indicate the potential for further adjustments of the clinical scan protocol aiming at a further reduction of the radiation dose.

## Figures and Tables

**Table 1:** Body mass indices (BMI, kg/m^2^); mean ± standard deviation (SD); IQL = image quality level. Mean BMI was highest in the patient group scanned with IQL 50 and lowest in the patient group scanned with IQL 60; differences in BMI between patient groups were not statistically significant.

	IQL 70			IQL 60			IQL 50			Stat. Test
	N	Mean	SD	N	Mean	SD	N	Mean	SD	
**Total**	31	**27.52**	±5.10	31	**25.89**	±5.10	36	**28.85**	±6.19	*p* = 0.097
**BMI ≤ 30**	23	**25.14**	±3.21	25	**24.08**	±3.68	23	**25.22**	±3.71	*p* = 0.437
**BMI > 30**	8	**34.36**	±2.64	6	**33.43**	±2.59	13	**35.29**	±4.03	*p* = 0.774
**Male**	15	**27.39**	±4.52	13	**27.10**	±3.92	24	**28.49**	±4.95	*p* = 0.632
**Female**	16	**27.64**	±5.74	18	**25.02**	±5.76	12	**29.58**	±8.34	*p* = 0.161

**Table 2:** Radiation dose parameters (Subgroups); mean ± standard deviation (SD); IQL = image quality level; CTDI = computed tomography dose index; DLP = dose length product; SSDE = size-specific dose estimate; † and ‡ indicate pairs with *p*-values < 0.05 in the post hoc test.

	IQL 70		IQL 60		IQL 50		Stat. Test
	Mean	SD	Mean	SD	Mean	SD	
**CTDI_vol_** (mGy)							
**Total**	†‡ **3.82**	±1.22	† **3.17**	±0.83	‡ **3.11**	±0.98	*p* = 0.011
**BMI ≤ 30**	† **3.39**	±0.78	**2.91**	±0.66	† **2.70**	±0.76	*p* = 0.008
**BMI > 30**	† **5.04**	±1.49	**4.25**	±0.60	† **3.85**	±0.89	*p* = 0.050
**Male**	**3.52**	±0.74	**3.38**	±0.70	**3.10**	±0.88	*p* = 0.236
**Female**	**4.10**	±1.52	**3.02**	±0.91	**3.14**	±1.18	*p* = 0.065
**DLP** (mGy*cm)							
**Total**	**158.16**	±57.98	**128.90**	±42.36	**130.00**	±45.75	*p* = 0.030
**BMI ≤ 30**	† **138.26**	±33.68	**117.40**	±33.48	† **110.35**	±32.55	*p* = 0.033
**BMI > 30**	**215.38**	±76.17	**176.83**	±44.36	**164.77**	±45.97	*p* = 0.199
**Male**	**144.73**	±37.82	**140.69**	±41.20	**131.63**	±43.16	*p* = 0.614
**Female**	**170.75**	±70.99	**120.39**	±42.25	**126.75**	±52.43	*p* = 0.044
**Effective Dose** (mSv)							
**Total**	**2.43**	±0.99	**2.02**	±0.67	**1.97**	±0.72	*p* = 0.046
**BMI ≤ 30**	† **2.12**	±0.58	**1.85**	±0.53	† **1.65**	±0.46	*p* = 0.028
**BMI > 30**	**3.33**	±1.36	**2.75**	±0.73	**2.54**	±0.76	*p* = 0.304
**Male**	**2.02**	±0.54	**2.01**	±0.60	**1.88**	±0.62	*p* = 0.739
**Female**	**2.81**	±1.16	**2.03**	±0.73	**2.14**	±0.90	*p* = 0.066
**SSDE** (mGy)							
**Total**	† **4.57**	±0.84	‡ **4.22**	±0.80	†‡ **3.53**	±0.70	*p* < 0.001
**BMI ≤ 30**	† **4.31**	±0.59	‡ **4.01**	±0.74	†‡ **3.31**	±0.70	*p* < 0.001
**BMI > 30**	† **5.32**	±1.04	‡ **5.11**	±0.19	†‡ **3.91**	±0.54	*p* < 0.001
**Male**	† **4.33**	±0.53	‡ **4.55**	±0.54	†‡ **3.51**	±0.71	*p* < 0.001
**Female**	† **4.80**	±1.02	**3.99**	±0.89	† **3.56**	±0.71	*p* = 0.003

**Table 3:** Signal-to-noise ratio (SNR) and image noise measured in muscular tissue and subcutaneous fat; mean ± standard deviation (SD); IQL = image quality level; † indicates pairs with *p*-values < 0.05 in the post hoc test.

	IQL 70		IQL 60		IQL 50		Stat. Test
	Mean	SD	Mean	SD	Mean	SD	
**Signal-to-Noise Ratio (SNR)**							
**Muscle**	† **6.20**	±1.52	**6.14**	±1.22	† **5.39**	±1.25	*p* = 0.023
**Fat**	**13.33**	±3.27	**13.92**	±3.30	**12.20**	±2.88	*p* = 0.079
**Image Noise**							
**Muscle**	**9.13**	±1.99	**8.87**	±1.65	**9.91**	±1.77	*p* = 0.052
**Fat**	**8.57**	±2.25	**8.20**	±1.83	**9.24**	±1.86	*p* = 0.099

**Table 4:** Qualitative image analysis rated by three radiologists; mean ± standard deviation (SD); IQL = image quality level.

	IQL 70		IQL 60		IQL 50		Stat. Test
	Mean	SD	Mean	SD	Mean	SD	
**Diagnostic confidence**	**4.44**	±0.91	**4.48**	±0.94	**4.47**	±0.83	*p* = 0.653
**Overall** **image quality**	**4.19**	±0.86	**4.28**	±0.94	**4.07**	±1.00	*p* = 0.311
**Image noise**	**3.85**	±0.74	**3.84**	±0.83	**3.76**	±0.82	*p* = 0.643
**Image sharpness**	**4.22**	±0.82	**4.35**	±0.82	**4.17**	±0.88	*p* = 0.269

## Data Availability

The data are available from the corresponding author upon reasonable request.
